# Impact of Amoxicillin Shortage on Pediatric Antibiotic Prescriptions in Primary Care †

**DOI:** 10.3390/antibiotics14030313

**Published:** 2025-03-18

**Authors:** Federica Pagano, Giulio De Marco, Benedetta Trojano, Chiara Amato, Maria Micillo, Gaetano Cecere, Alfredo Guarino, Andrea Lo Vecchio

**Affiliations:** 1 Department of Translational Medical Sciences, Section of Pediatrics, University of Naples Federico II, Via Pansini 5, 80131 Naples, Italyalfguari@unina.it (A.G.); 2 PhD National Program in One Health Approaches to Infectious Diseases and Life Science Research, Department of Public Health, Experimental and Forensic Medicine, University of Pavia, 27100 Pavia, Italy; 3 U.O. Materno Infantile, ASL Napoli 1 Centro, Distretto Sanitario 28, 80145 Naples, Italy

**Keywords:** amoxicillin, antimicrobial stewardship, primary care, drug accessibility, shortage

## Abstract

**Background/Objectives:** A previous study settled in the Campania Region (Southern Italy) has proven the effectiveness of a multifaceted antimicrobial stewardship program in reducing prescription rates and use of broad-spectrum molecules in the Primary Care setting. Since autumn 2022, the amoxicillin shortage has been reported at a national level, and respiratory pathogens resurged in children after the easing of COVID-19 pandemic restrictions. We aimed to assess the impact of amoxicillin shortage on antimicrobial prescription patterns and quality indexes in the same setting as the past AMS campaign. **Methods:** We conducted a retrospective review of antibiotic prescriptions in a primary care pediatric practice, focusing on amoxicillin, amoxicillin-clavulanate, third-generation cephalosporins, macrolides, and quinolones. To assess drug accessibility, we monitored antibiotic availability in pharmacies within the same healthcare district. We then analyzed monthly prescription rates per 100 consultations in relation to drug availability patterns and calculated the amoxicillin/amoxicillin-clavulanate index and the Access/Watch index as quality indicators. **Results:** From November 2022 to May 2023, 90% of the surveyed pharmacies reported an amoxicillin shortage lasting 5 to 7 months. Concomitantly, we observed a significant shift in the prescription pattern for amoxicillin-clavulanate (3.53 to 13.82; *p* = 0.009) and third-generation cephalosporins (2.45 to 4.83; *p* = 0.026), that resulted in a decline of the amoxicillin/amoxicillin-clavulanate index (1.38 to 0.56; *p* = 0.009). **Conclusions:** The lack of amoxicillin could have led to increased prescriptions of second-line antibiotics in Italian regions, reverting the effect of successful stewardship measures.

## 1. Introduction

Antimicrobial stewardship (AMS) is essential in regions with high antibiotic resistance rates, such as the Campania region in Southern Italy [1,2]. In recent years (2016–2019), the regional health committee implemented a multifaceted AMS program addressed to primary care physicians, which included the mandatory requirement of ICD-9 code for each antibiotic prescription, the establishment of a technical committee for surveillance on hospital-acquired infection risk and AMR, the spreading of guidelines for the implementation of AMS programs and regional protocols on antibiotic therapy and an e-learning course on antibiotic therapy and AMS addressed to healthcare professionals (biologists, chemists, pharmacists, nurses, medical doctors, obstetricians, and veterinarians). A previous study conducted during this period proved the effectiveness of these measures in reducing the prescription rates by Primary Care Pediatricians (PCPs) [3]. Since late 2022, an amoxicillin shortage has been described in several Western countries [4]. However, available studies present heterogeneous outcome measures and report short follow-up periods, lacking a link to the local ongoing AMS projects. Concomitantly, communicable respiratory pathogens surged in children after the easing of COVID-19 pandemic restrictions [5,6,7,8].

The present study aimed to assess the impact of amoxicillin shortage on antimicrobial prescription patterns and quality indexes in the same setting as the above-mentioned AMS campaign, whose effectiveness was already proven [3].

## 2. Results

### 2.1. Availability of First-Line Antimicrobials

**Figure 1:**
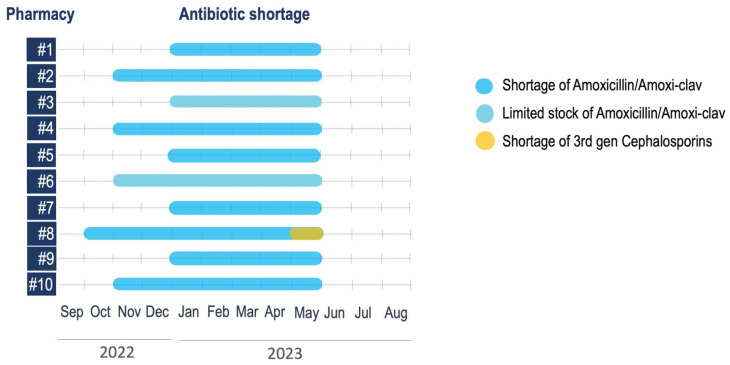
The availability of amoxicillin/amoxicillin-clavulanic acid and III-generation cephalosporins among ten pharmacies surveyed between September 2022 and August 2023.

Ten territorial pharmacies within the PCPs-associated practice Sanitary District were enrolled in a telephonic survey aimed at investigating the availability of oral formulations of amoxicillin, amoxicillin-clavulanate, 3rd generation cephalosporins, and macrolides. Eight out of ten pharmacies reported experiencing a shortage of amoxicillin or amoxicillin-clavulanate suspensions or tablets both at the time of the interview and in the previous months, while two indicated having limited stock. None of the participating pharmacies reported a shortage of macrolides, while only one lacked third-generation cephalosporins. Most pharmacies (90%, Figure 1) reported an antibiotic shortage lasting 5 to 7 months (from November 2022 to May 2023), and only one reported 8 months (from October 2022 to May 2023) [9].

### 2.2. Trends in Antibiotic Prescription

During the study period (2016–2023), a total of 15,388 antibiotic prescriptions were documented within the PCPs-associated practice involved in the study (mean = 1935 prescriptions/year). A relevant change in the distribution of different molecules was observed. In 2016, we registered 3107 prescriptions overall: 9.95% were amoxicillin prescriptions (n = 309), 44.90% amoxicillin-clavulanate (n = 1395), 15,58% macrolides (n = 484), and 25.39% 3rd generation cephalosporins (n = 789). In 2019, at the end of AMS interventions, the analysis of 2064 prescriptions showed 33.33% amoxicillin prescriptions (n = 688), 33.43% amoxicillin-clavulanate (n = 690), 16.47% macrolides (n = 340), and 13.28% 3rd generation cephalosporins (n = 273).

A nested analysis of 1978 prescriptions performed in the semester from November 2022 to April 2023 (during the antibiotic shortage) demonstrated a significant decrease in amoxicillin (n = 352, 17.79%) and macrolides (n = 153, 7.73%) usage and a relevant increase in the prescription of amoxicillin-clavulanate (n = 1032, 52.17%) and 3^rd^-generation cephalosporins (n = 335, 16.93%). Detailed prescription data are presented in Table 1.

**Figure 2:**
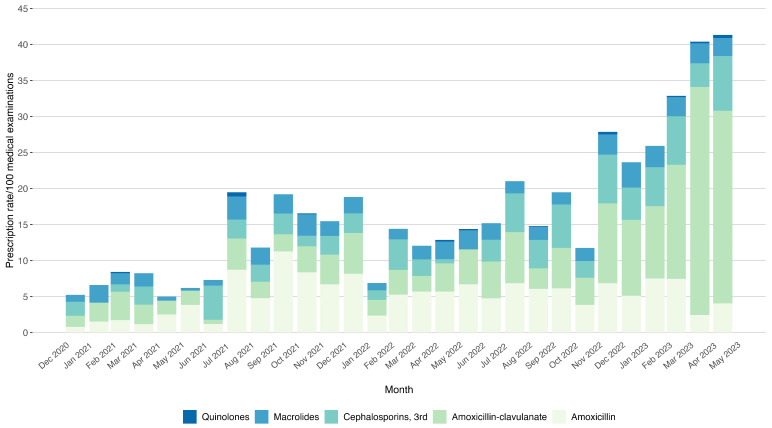
Antibiotic prescription distribution and classes by PCPs (January 2021–May 2023).

Following the second wave of the COVID-19 pandemic (2021–2023), concomitantly with a loosening of non-pharmaceutical preventive measures (masks, social distancing, and limitations in outdoor activities), we observed a progressive increase in the monthly prescription rate per 100 medical consultations from 5.43 in January 2021 to 41.33 in May 2023 (R^2^ = 0.634, *p* < 0.001). Prescription rates for single antibiotic molecules also varied: amoxicillin rate increased from 0.78 to 4.05 (R^2^ = 0.246, *p* = 0.04) and amoxicillin-clavulanate from 1.55 to 26.75 (R^2^ = 0.891, *p* = 0.10). In the same period, we observed a significant increase in the rates of 3rd-generation cephalosporins (from 1.94 to 7.60; R^2^ = 0.483, *p* = 0.02). Macrolides and quinolones did not show significant changes. The variations in the prescription rates are illustrated in Figure 2.

When comparing the six months following the shortage (November 2022–April 2023) to the corresponding semester in the previous year, the mean prescription rate per 100 consultations rose significantly from 15.48 to 26.98 (*p* = 0.041). Notably, there was a significant shift in the prescription pattern for amoxicillin-clavulanate (3.53 to 13.82; *p* = 0.009) and third-generation cephalosporins (2.45 to 4.83; *p* = 0.026). However, no significant change in the amoxicillin prescription rate was observed (6.09 to 5.54; *p* = 0.699), probably due to the shortage [9].

In the interrupted time series (ITS) analysis of specific prescription rates before and after the start of amoxicillin shortage, only amoxicillin-clavulanic acid had a significant increase, considering both the period immediately after the shortage and the following months [Appendix A, Table A1].

### 2.3. Trends in Prescription Quality Indexes

**Figure 3:**
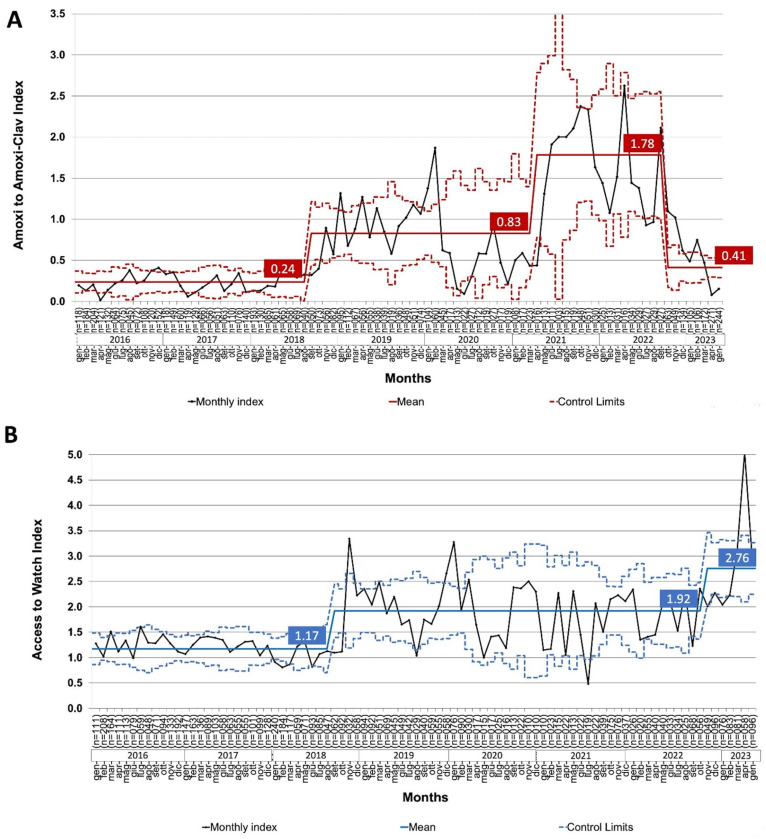
The trend of amoxicillin/amoxicillin-clavulanate (**A**) and Access/watch (**B**) indexes. Black dots connected by a solid black line represent monthly indexes. The dashed line represents the 95% confidence interval. The solid red and blue lines represent the average value of amoxicillin/amoxi-clav and Access/Watch index, respectively (also reported in the boxes).

After AMS interventions, the mean amoxicillin/amoxicillin-clavulanate index gradually increased from 0.24 to 1.78 but suddenly dropped to 0.15 in May 2023, with a significant difference in the six-month mean before and after the shortage (1.38 to 0.56; *p* = 0.009) [9]. The run charts in Figure 3 depict the trend of the amoxicillin/amoxicillin-clavulanate index and Access/Watch index since the beginning of our observation. In parallel with the implementation of the AMS bundle, the amoxicillin/amoxicillin-clavulanate index gradually improved from 0.24 (2016) to 1.78 (2022) [9]. After the beginning of the amoxicillin shortage (November 2022), there was a sudden downshift, and the index relapsed to 0.41. On the other hand, the trend of the Access/Watch index continued to rise both before and after the shortage, starting from 1.17 (2016) and reaching a value of 2.76 (2023).

**Figure A1:**
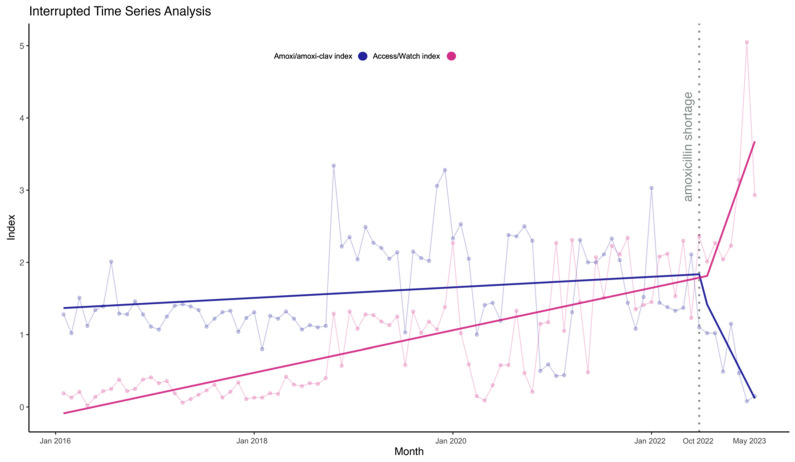
ITS analysis of amoxicillin/amoxicillin-clavulanic acid and Access/Watch indexes (2016–2023).

The Interrupted time series analysis of the same indexes (2021–2023) demonstrates that starting from November 2022, the amoxi/amoxi-clav index showed a temporary slope change (β = −0.78), leading to a level and direction change (β = −0.15; R^2^ = 0.221), while the Access/Watch index showed a slope change and continued to rise more rapidly after amoxicillin shortage (β = 0.29; R^2^ = 0.714) [Appendix B, Figure A1, and Table A2].

## 3. Discussion

Antimicrobial Stewardship interventions implemented in various pediatric care settings (inpatient, emergency department, and PC) have been effective in improving overall prescription rates and adherence to guidelines’ recommendations. However, evidence regarding the reduction in the use of broad-spectrum antibiotics in PC settings remains weak [10].

Due to the high rate of antibiotic resistance resulting from frequent and inappropriate use of antibiotics in Southern Italy, the Campania region’s healthcare department introduced a multifaceted AMS program in 2016 [3]. This program included the development of an AMS task force, the creation and implementation of local guidelines, the dissemination of web-based educational interventions, the active monitoring of antibiotic consumption and resistance patterns, and the introduction of mandatory ICD-10 code declarations for prescriptions. We previously demonstrated that the application of such a bundle of AMS interventions was associated with a significant reduction of overall antibiotic prescription rates and the use of broad-spectrum molecules such as amoxicillin-clavulanate, 3rd cephalosporins and azithromycin by PCPs [3]. This change in prescription resulted in a progressive improvement of the amoxicillin/amoxicillin-clavulanate index as well as the access-watch index according to WHO AWaRe classification [11].

However, from October 2022 to May 2023, we witnessed a nationwide antibiotic shortage that had an impact on prescriptions. In this scenario, our data demonstrates that the availability of first-line antibiotics is a major determinant of prescription quality. The local shortage of oral formulations of amoxicillin has led to a substantially increased prescription rate for amoxicillin-clavulanate and 3rd generation cephalosporins. The overall increase in prescription rates could reflect the surge in communicable diseases following the COVID-19 pandemic and the de-prioritization of AMS interventions during the pandemic. Furthermore, the disparity in the trajectories of the amoxicillin/amoxicillin-clavulanate and Access/Watch indexes can be attributed to the substantial surge in monthly amoxicillin-clavulanate (included in Watch category) prescriptions, which overshadowed the gains seen with third-generation cephalosporins.

A chronic shortage of amoxicillin and amoxicillin-clavulanic acid has also been described in Japan since May 2023 due to increased demand following a rise in antibiotic prescriptions for respiratory and gastrointestinal infections. Differently from our results, in a large cohort of pediatric outpatients, Otsubo et al. registered a fall in the Access/Watch index after the shortage as a consequence of increased prescriptions of cephalosporins and other broad-spectrum antimicrobials, while the proportion of amoxicillin and amoxicillin-clavulanic acid both decreased [12].

Our results are more comparable to those published from other surveillance networks in the USA on outpatient prescriptions for pediatric acute respiratory infections [13,14].

Indeed, the shortage registered in the USA in the autumn of 2022 impacted amoxicillin more than amoxicillin-clavulanic acid formulations, allowing a significative switch in prescriptions from the first to the latter (besides other molecules, e.g., cephalosporines) for upper respiratory tract infections and otitis media.

Although in our cohort of pharmacies, both formulations seemed equally lacking, only amoxicillin prescriptions were significantly impacted, while amoxi-clavulanate continued to rise after the shortage. Several factors could have contributed to this discrepancy. Our survey enrolled a limited sample of pharmacies, and most of them declared weekly fluctuations in the availability of amoxicillin-clavulanic acid. This could suggest that pediatricians preferred to prescribe the second molecule, believing the patients would have a better chance of obtaining it. Nonetheless, our data include only information on prescriptions made by PCPs. We could not track the effective consumption, nor did we know how many prescribed therapies were successfully accomplished. Although we surveyed pharmacies located in the same sanitary district of PCPs’ practice, it cannot be excluded that our population could have been drawn to pharmacies outside this area to source the lacking antibiotics. Finally, as the pharmacies self-reported the shortages by consulting the records and procurement requests, recall bias cannot be excluded.

Our study has other limitations. Firstly, the lack of data about the diagnosis linked to the antibiotic prescriptions, recurrence, and risk factors of patients receiving antibiotics does not allow for the analysis of appropriateness. However, an 8-year continuous monitoring of the same associated pediatric practice may attenuate internal variability and risk of bias. Secondarily, the time that occurred since the end of AMS interventions in the Campania Region could play a role in a return to baseline prescribing practices [15,16].

In our opinion, this should be a limited phenomenon in our cohort as macrolides prescription rates, which were one of the main targets of the AMS campaign, didn’t rebound and were very limited both pre- and post-shortage.

The findings suggest that authorities and stakeholders should implement actions to guarantee the availability of essential medicines. Possible strategies include monitoring supply and demand, tracking critical medications, promoting international collaborations to improve production and distribution, adopting flexible regulations to ensure quick access to medicines (such as extending shelf life or fast-tracking alternatives), and generally increasing public awareness about antibiotics consumption.

## 4. Materials and Methods

### 4.1. Study Design

We conducted a two-phase study, consisting of a first retrospective cohort study (January 2016–May 2023) on the antimicrobial prescription data of an associate practice of three PCPs located in Naples, Campania Region, Southern Italy, completed by a cross-sectional survey investigating the availability of antibiotic molecules among local pharmacies.

### 4.2. Data Collection

#### 4.2.1. Antimicrobial Consumption

In line with the AMS protocol and methodology used in a previous study [3], data regarding outpatient antimicrobial prescriptions were continuously collected using the study manager software Kappamed (version 12.4.30). This study is part of a more extensive Antimicrobial Stewardship Program promoted by the Regional Committee for Antimicrobial Stewardship and counteracts the Antimicrobial Resistance that has been ongoing since 2016. The present analysis, which comes from the collaboration with family pediatricians involved in the AMS program, did not require formal approval by the Ethical Committee, as it does not involve any personal data of the patients or caregivers and only reports aggregate data concerning antibiotic prescriptions as a part of an active monitoring system ongoing on the regional territory since the implementation of the AMS programs. This collaboration resulted in a previous publication on the same topic [3]. 

In addition, parents or legal guardians provided informed consent to the processing of the patient’s personal data at the time of the first medical consultation, according to local procedures and to the Declaration of Helsinki.

The study population consisted of all the patients assigned to the PCPs during the study period. The extracted data included the number of medical consultations and antibiotic prescriptions (date and molecule) performed by the PCPs monthly and were registered in a database created with Microsoft Excel, released 16.90.2. No sensible information about patients was included in the dataset.

Consistent with the last Campania Region report on local antimicrobial consumption and resistance, we monitored the prescription of amoxicillin, amoxicillin-clavulanate, 3^rd^-generation cephalosporins, macrolides, and quinolones, whose resistance rates are the most elevated respecting the European average [17]. Aminoglycosides and carbapenems were excluded from the data collection as they are sparsely used in outpatient settings in children.

#### 4.2.2. Antimicrobial Availability

To substantiate the shortage of amoxicillin within the area, we conducted a cross-sectional survey through telephone interviews in May 2023 among the pharmacies situated within the Sanitary District associated with the (PCPs) practices. A verbal informed consent was obtained from the pharmacists involved in the telephonic survey. Pharmacists were asked to declare the availability of oral formulations of amoxicillin, amoxicillin-clavulanate, 3rd-generation cephalosporins, macrolides, and quinolones. Data were declared by consulting the records and procurement requests.

The survey included nine questions:

(1) Is there any supply of amoxicillin (oral suspension-based formulations) in your pharmacy?
(2) Is there any supply of amoxicillin (tablet-based formulations) in your pharmacy?
(3) Is there any supply of amoxicillin-clavulanic acid (oral suspension-based formulations) in your pharmacy?
(4) Is there any supply of amoxicillin-clavulanic acid (tablet-based formulations) in your pharmacy?
(5) Is there any supply of cefixime (oral suspension) in your pharmacy?
(6) Is there any supply of cefaclor (oral suspension) in your pharmacy?
(7) Is there any supply of macrolides (either azithromycin or clarithromycin, oral suspension) in your pharmacy?
(8) Is there any supply of ciprofloxacin (oral suspension) in your pharmacy?
(9) Was there any supply of amoxicillin (oral suspension) in your pharmacy in the last week?
(10) When did supply difficulties start in your pharmacy? (If supply difficulties were declared for amoxicillin and/or amoxicillin-clavulanic acid oral suspension).

### 4.3. Data Analysis and Outcome Measures

The primary outcome was the trend of the amoxicillin/amoxicillin-clavulanate index and the Access/Watch index, which were selected as indicators of prudent use of antimicrobials according to the European Commission’s recommendations [18]. The Access/Watch index was calculated according to the AWaRe classification by the World Health Organization (WHO) [11].

The secondary outcomes were the monthly overall prescription rate and specific prescription rates of amoxicillin, amoxicillin-clavulanate, 3rd-generation cephalosporins, macrolides, and quinolones. 

All prescription rates were calculated as the monthly number of prescriptions standardized for 100 medical consultations to minimize the seasonal variability and the differences in the number of patients assisted by each pediatrician. All the outcomes were evaluated over the study period and correlated with the start of the amoxicillin shortage. In order to further exclude the effect of seasonal variability, an additional comparison between the prescription rates of the winter season following the shortage (November 2022–April 2023) and the same semester of the previous year was performed.

Proportions were expressed as percentages (%). The variation of the outcomes during the study period was analyzed with a linear regression model. A *p*-value of less than 0.05 was considered significant. A run chart was constructed and interpreted to evaluate changes in the central tendency of monthly the amoxicillin/amoxicillin-clavulanate and Access/watch indexes using the following criteria: a shift in the process or too many data points in a run (8 or more consecutive points above or below the median) and a trend (6 or more consecutive points all increasing or decreasing) [19,20]. The correlation between the amoxicillin shortage and changes in the trend of outcome measures has been tested through Interrupted Time Series (ITS) analysis [21]. The two sub-periods (pre- and post-shortage) were compared with a segmented linear regression, with only a breakpoint placed in November 2022. Four variables were considered in the analysis: the overall time elapsed in the study period (in months), a dummy variable indicating the pre-shortage period (coded 0) or the post-shortage period (coded 1), the time elapsed since the start of shortage to the end of the study period (in a month), the outcome at each time unit. The analysis was checked for autocorrelation (Durbin–Watson test), model fit (residual diagnostics with Shapiro–Wilk test), and model robustness (model ARIMA).

The statistical analysis and data visualization were performed using IBM SPSS Statistics, released 29.0.1.0, and R with RStudio interface, released 2024.04.1+748.

## 5. Conclusions

Most AMS interventions typically focus on ensuring adequate indications, dosages, and durations of treatment, as well as implementing measures to reduce inappropriate prescriptions (e.g., vaccination, rapid diagnostics), improve appropriateness, and enhance caregivers’ literacy on fundamental antibiotic therapies. However, our data underscore the crucial role of access to essential medicines in achieving these outcomes. These findings should prompt authorities and stakeholders to implement interventions to ensure the availability of essential medicines (e.g., monitoring supply-demand, tracing critical medicines, fostering international supply partnerships to increase production and distribution, adopting regulatory flexibilities to allow timely access to medicines (including extending shelf-life or quickly authorizing alternatives), and raising public awareness about prudent antibiotic use).

## Figures and Tables

**Table 1:** Antibiotic prescription distribution and classes.

Year	Amoxicillin	Amoxicillin-Clav.	Azithromycin	Clarithromycin	Cephalosporins, 3rd	Quinolones	Overall Yearly Prescriptions
2016	309 (9.95)	1395 (44.9)	118 (3.8)	366 (11.78)	789 (25.39)	8 (0.26)	3107
2017	256 (9.48)	1237 (45.83)	106 (3.93)	410 (15.19)	614 (22.75)	11 (0.41)	2600
2018	289 (12.04)	962 (40.07)	95 (3.96)	434 (18.08)	474 (19.74)	22 (0.92)	2401
2019	688 (33.33)	690 (33.43)	31 (1.5)	309 (14.97)	274 (13.28)	7 (0.34)	2064
2020	360 (32.94)	382 (34.95)	38 (3.48)	128 (11.71)	146 (13.36)	2 (0.18)	1093
2021	427 (43.52)	230 (23.44)	70 (7.13)	95 (9.68)	153 (15.59)	5 (0.5)	981
2022	540 (34.77)	477 (30.71)	44 (2.83)	150 (9.65)	335 (21.57)	8 (0.51)	1553
2023 ^1^	269 (16.92)	898 (56.51)	42 (2.64)	100 (6.29)	272 (17.11)	8 (0.5)	1589
Total							15,388

Prescriptions are expressed as numbers. Percentages in brackets are calculated over the yearly total prescriptions (%). ^1^ January–May 2023.

**Figure A2:**
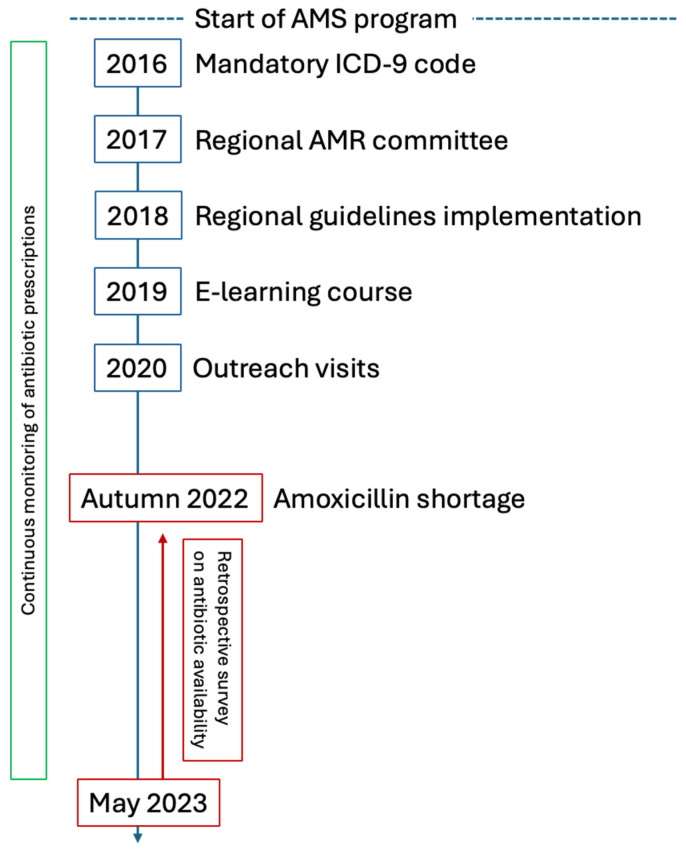
Methodology of the study and institutional framework. The flowchart illustrates the methodology of the study and the actions implemented within the Regional AMS program in the same setting (2016–2023).

## Data Availability

The corresponding author will make the data available upon reasonable request.

## Abbreviations

The following abbreviations are used in this manuscript:

AMS	Antimicrobial stewardship
AMR	Antimicrobial resistance
ICD-10	International Classification of Diseases—10th edition
PC	Primary Care
PCP	Primary Care Pediatrician
ITS	Interrupted Time Series

## Appendix A

**Table A1:** Interrupted time series (ITS) analysis of prescription rates. Coefficients are reported for the overall period (Month), for the month following shortage (Shortage), and for the period following shortage (Month after shortage).

	Overall			Amoxicillin			Amoxicillin-Clav.			Cephalosporins, 3rd			Macrolides			Quinolones		
Predictors	β	95%CI	*p*	β	95%CI	*p*	β	95%CI	*p*	β	95%CI	*p*	β	95%CI	*p*	β	95%CI	*p*
(Intercept)	−48.62	−68.57–−28.67	**<0.001 ***	−12.52	−29.64–4.59	0.144	−6.98	−19.56–5.60	0.264	−7.03	−15.05–0.99	0.083	−0.81	−7.00–5.39	0.791	0.07	−0.64–0.79	0.832
Month	0.88	0.62–1.14	**<0.001 ***	0.24	0.01–0.48	**0.044 ***	0.15	−0.03–0.32	0.100	0.13	0.02–0.24	**0.023 ***	0.04	−0.05–0.12	0.382	−0.00	−0.01–0.01	0.976
Shortage				−1.66	−7.22–3.89	0.543	−5.75	−10.64–−0.86	**0.023 ***	−0.01	−3.16–3.13	0.993	0.19	−1.49–1.87	0.818	−0.08	−0.35–0.20	0.577
Month (after shortage)				−0.41	−1.61–0.79	0.484	3.98	2.98–4.97	**<0.001 ***	0.25	−0.39–0.89	0.431	0.02	−0.38–0.41	0.932	0.05	−0.01–0.10	0.111
R^2^	0.634			0.246			0.891			0.483			0.254			0.182		

* = *p* value < 0.05.

## Appendix B

**Table A2:** ITS analysis of amoxicillin/amoxicillin-clavulanic acid and Access/Watch indexes (2021–2023). Coefficients are reported for the overall period (Month), for the month following shortage (Shortage), and for the period following shortage (Month after shortage).

	Amoxi/Amoxi-Clav Index			Access/Watch Index		
Predictors	β	R^2^	*p*	β	R^2^	*p*
(Intercept)	1.27	0.226	<0.001 *	−0.11	0.714	0.450
Month	0.01		0.082	0.02		<0.001 *
Shortage	−0.78		0.248	−0.29		0.567
Month (after shortage)	−0.15		0.269	0.29		0.009 *

* = *p* value < 0.05.
